# EDITORIAL COMMENT: LAPAROSCOPIC RADICAL NEPHRECTOMY WITH INFERIOR VENA CAVA THROMBECTOMY: HIGHLIGHT OF KEY SURGICAL STEPS

**DOI:** 10.1590/S1677-5538.IBJU.2015.0080.1

**Published:** 2016

**Authors:** Philippe E. Spiess

**Affiliations:** 1 Department of Urologic Oncology. H. Lee Moffitt Cancer Center, Tampa, Florida, USA

In this video by Sim et al. (1), the authors nicely depict how laparoscopic surgery can be employed to tackle locally advanced renal tumors with venous vascular extension. The authors are to be congratulated on their elegant approach to such a case resulting in minimal blood loss, enhanced perioperative recovery, and most importantly strictly adhering to the essential principles of surgical oncology with complete tumor eradication. This being said, I would like to emphasize the last statement made by the authors in the their abstract which is that such an approach should be conducted in only highly selected cases at centers of excellence. I think low level IVC tumor thrombi (Mayo classification level 1 and 2) maybe appropriate to address in this manner in specific instances but tumor thrombi exhibiting intrahepatic or intracardiac extension (level 3 and 4, respectively) should be empirically approached using an open approach although some recent reports have raised the potential of robotic minimally invasive surgery in very highly selected cases. One can never forget the inherent morbidity associated with such high level IVC tumor thrombi cases, where the margin for error is infinitely small. Rapidly evolving technology will continually push the envelope as to how we perform surgery but the onus lies upon us as treating surgeons to always place the safety and wellbeing of our patients as the unwavering Hypocratic oath we will always adhere to.

*Philippe E. Spiess, MD* *Assistant Professor of Urologic Oncology* *H. Lee Moffitt Cancer Center* *Tampa, Florida, USA* *E-mail: philippe.spiess@moffitt.org*
